# Clinical Experience Over 15 Years with the B-Lynch Compression Suture Technique in the Management of Postpartum Hemorrhage

**DOI:** 10.1055/s-0041-1735228

**Published:** 2021-10-20

**Authors:** Gilberto Nagahama, Henri Augusto Korkes, Nelson Sass

**Affiliations:** 1 Department of Obstetrics, Hospital Maternidade Escola de Vila Nova Cachoeirinha, São Paulo, SP, Brazil; 2 Department of Obstetrics and Gynecology, Faculty of Medicine, Pontifícia Universidade Católica de São Paulo, São Paulo, SP, Brazil; 3 Department of Obstetrics, Escola Paulista de Medicina, Universidade Federal de São Paulo, São Paulo, SP, Brazil

**Keywords:** postpartum hemorrhage, uterine atony, suture techniques, maternal mortality, surgical hemostasis, hemorragia pós-parto, atonia uterina, técnicas de sutura, mortalidade materna, hemostasia cirúrgica

## Abstract

**Objective** To describe the clinical experience with the B-Lynch technique in the management of postpartum hemorrhage as well as the factors related to the indication of the technique and to present the success rates of the application of the B-Lynch technique.

**Methods** Observational, retrospective, cross-sectional, and analytical study. Patient data was obtained through the study of medical records. The study population comprised of patients who underwent hemostatic suture using the B-Lynch technique, including 104 patients within the period from January 1, 2005, to December 31, 2019.

**Results** Of the total of 104 patients, 82.7% did not present any complications. Blood transfusion and intensive care unit admission were the most prevalent complications, with 13.5% and 15.4%, respectively. Only 1% of the patients had puerperal and surgical site infections. The factors most related to the application of the technique were the presence of previous cesarean section (30.8%), use of oxytocin (16.3%), and preeclampsia (11.6%). Puerperal hysterectomy was performed in 4.8% of the patients due to failure of the method.

**Conclusion** The clinical experience with the B-Lynch technique was satisfactory since it presented few complications, with excellent results in hemorrhagic control. Previous cesarean section, the use of oxytocin, and preeclampsia stood out as factors related to the indication of the application of the technique, and the success rate in controlling postpartum hemorrhage was 95.2%.

## Resumo

**Objetivo** Descrever a experiência clínica com a técnica de B-Lynch no manejo da hemorragia pós-parto e os fatores relacionados à indicação da técnica bem como apresentar as taxas de sucesso da aplicação da técnica de B-lynch.

**Métodos** Estudo observacional, retrospectivo, de corte transversal e analítico. Os dados foram obtidos por estudo de prontuário. A população do estudo foi constituída de pacientes submetidas à sutura hemostática com a técnica de B-Lynch, sendo incluídas 104 pacientes dentro do período de 01 de janeiro de 2005 a 31 de dezembro de 2019.

**Resultados** Do total de 104 pacientes, 82,7% não apresentaram qualquer complicação. A transfusão de sangue e a internação na UTI foram as complicações mais prevalentes, com 13,5% e 15,4%, respectivamente. Apenas 1% teve infecção puerperal e do sítio cirúrgico. Os fatores mais relacionados com a aplicação da técnica foram a presença de cesárea anterior (30,8%), uso de ocitocina (16,3%) e pré-eclâmpsia (11,6%). A histerectomia puerperal foi realizada em 4,8% das pacientes por falha do método.

**Conclusão** A experiência clínica com a técnica de B-Lynch foi satisfatória, pois apresentou poucas complicações, com excelentes resultados no controle hemorrágico. A cesárea anterior, o uso de ocitocina e a pré-eclâmpsia se destacaram como fatores relacionados à indicação da aplicação da técnica. A taxa de sucesso avaliada foi de 95,2%.

## Introduction

Maternal mortality (MM) is a tragic event of worldwide proportions. Worldwide, it is estimated that 300,000 maternal deaths occur every year according to the World Health Organization (WHO), 94% of which occur in poor or developing countries. Note that the majority could have been avoided. 1

Maternal mortality has been declining globally, with significant differences among several countries. Effective compliance with strategies for its reduction provided a greater understanding of the social causes of MM, such as the role of education, income distribution, and place of birth. 2 3

In Brazil, according to the WHO, the national MM ratio (MMR) was 60 deaths in 2017. Compared to the year 1990, which had a MMR of 104 deaths, a significant reduction of 42.3% occurred in this period. However, when we analyze countries such as Japan and the United States of America, which presented, respectively, MMRs of 5 and 19 deaths in the same period, it is clear how much progress needs to be made so that women's health can reach acceptable coefficients in our country. 2

Postpartum hemorrhage (PPH) is responsible for 25 to 30% of MM worldwide. In Brazil, 13.3% of MM is due to antepartum and PPH. It is the second most important direct obstetric cause, and, unfortunately, it remains one of the most important causes of preventable MM. 1 4

Uterine atony (UA) is the most common cause of PPH, and it is responsible for ∼ 80% of all cases of this concerning pathology. 5 The traditional treatment of UA is established by a sequence of actions well defined by global protocols. In the case of therapeutic failure with the initial clinical pharmacological measures, other methods must be promptly applied to control uterine hemorrhage. Surgical methods such as selective arterial embolization, internal iliac or uterine artery ligation, and total or subtotal hysterectomy are presented as effective treatment options. 6 7 However, they show an excessively invasive nature, requiring longer execution time, and a long learning curve; consequently, they are associated with more comorbidities, such as greater volume of hemorrhage and risk of injury to adjacent structures, such as the ureter, intestines, and bladder. 7

The search for less invasive techniques easily reproducible and aiming at the possible preservation of fertility to avoid hysterectomies has brought about the so-called hemostatic suture techniques for the correction of atony conditions refractory to the initial measures. 8 9 10 11 12 13 14

Hemostatic sutures have been part of the therapy to control PPH for at least two decades. Several techniques have emerged over the years with the same purpose of controlling PPH after non-surgical therapeutic failure and preserving the patient from more complex surgeries with greater comorbidities, such as hysterectomy. 7 8 9

In Oxford, Christopher B-Lynch described a hemostatic compression suture technique, in 1997, in five cases of PPH secondary to UA which did not respond to the initial clinical-pharmacological treatment, thus introducing a new conservative alternative to surgeries with greater morbidity. 8 The application of the technique has been recommended worldwide, being included in the routine clinical practice for PPH in several protocols around the world. A systematic review showed 91.7% of success rate in controlling PPH. 9 10 14 15

It is important to document this experience in a clinical study aimed at analyzing the clinical experience with the B-Lynch compression suture technique in the management of PPH at the Hospital Maternidade Escola Vila Nova Cachoeirinha (HMEVNC) over 15 years, in addition to identifying the factors related to the indication for use of the technique. The success rates of the B-Lynch compression suture technique in controlling PPH were also analyzed.

## Methods

This is an observational, retrospective, cross-sectional, and analytical study. The study population consisted of patients who underwent uterine compression suture using the B-Lynch technique at HMEVNC within the period from January 1, 2005, to December 31, 2019. The number of patients included was defined by a convenience sample of 104 patients.

Regarding the use of the method, we state that the B-Lynch hemostatic suture was included in the clinical practice protocol of the HMEVNC as of 2005. This pioneering experience in Brazil was recorded in a national publication reporting a series of four cases of successfully controled PPH using the B-Lynch suture technique. 16 Since then, this experience was expanded to be incorporated into their routine clinical practices.

In our center, the indication for applying the B-lynch suture was exclusively due to UA related to several risk factors and management of UA is established by a sequence of actions. These actions initiated immediately after the diagnosis of PPH due to UA initially include bimanual uterine compression, described as the Hamilton maneuver concomitant with the use of uterotonics, with intravenous oxytocin being the first option. The ergot alkaloids should be applied if the initial therapy with oxytocin fails, observing the contraindication for hypertensive patients. Misoprostol, an analogue of type E1 prostaglandin, is the third drug in the therapeutic sequence. Intrauterine balloon tamponade is not available at our center. Therefore, if after pharmacological treatment fails to control PPH, we immediately apply the B-lynch suture, regardless of the parity or severity of uterine bleeding.

The document analysis to obtain the patient's data was performed on the study of medical records. These records were selected from a notebook available at the obstetric center for exclusive use to write down the full names and medical record of the patients who underwent the B-Lynch suture technique. Data was collected exclusively by the main researcher using a spreadsheet created with the software Microsoft Excel, version for Office 365 (Microsoft Corp., Redmond, WA, USA).

The selected analysis variables were grouped and defined as follows:

Pregnancy and childbirth variables:

1. Gestational age: defined in complete weeks at the time of delivery, counting by the date of the last menstrual period (LMP), if reliable, or by ultrasound, if LMP w unknown or unreliable.
2. Number of pregnancies: considered the sum of current and previous pregnancies, regardless of gestational age, vitality, or location.
3. Parity: defined as the number of deliveries whose newborn weighed ≥ 500 g or reached gestational age ≥ 20 weeks, regardless of the number of fetuses or outcomes.
4. Antenatal care: considered performed only for patients who attended six or more appointments. 17
5. Use of oxytocin to induce or conduct labor;
6. Previous cesarean section: 1 or more;
7. Preeclampsia: manifestation of hypertension after the 20th week of pregnancy associated with significant proteinuria or associated with systemic impairment or damage to the target organs (thrombocytopenia, liver dysfunction, renal failure, pulmonary edema, impending eclampsia, or eclampsia) 18 ;
8. Polyhydramnios: defined as an increase in the amount of amniotic fluid 19 ;
9. Placental abruption: defined as the premature separation of the normal-sited placenta from the uterus 20 ;
10. Fetal macrosomia: defined as birthweight greater than 4,000 g 21 ;
11. Grand multiparity: defined as five deliveries or more 22 ;

Postpartum variables

1. Blood transfusion: the process of transferring blood components, such as red blood cells, plasma, clotting factors, and platelets was based on hemodynamic instability with hemoglobin lower than 7 g/dL or coagulopathy.
2. Postpartum infection: defined as reproductive tract infection occurring after delivery. 23
3. Complications of the surgical wound: defined as an abnormality in the evolution of healing, such as dehiscence and surgical site infection.
4. Failure of the method: defined as the need for puerperal hysterectomy after application of the B-Lynch technique.
5. Puerperal hysterectomy: defined as a surgical procedure for removing the uterus by laparotomy when hemodynamic instability persists after failure of conservative hemostatic methods. 7

It is important to note that the data collected from the postpartum variables was restricted only to the patients' hospitalization period, considered a short period of observation.

The data was analyzed using descriptive methods to characterize the variables collected during the study. Categorical variables were analyzed using absolute and relative frequencies. Continuous variables were analyzed using medians. For qualitative variables, the ratios were also estimated with their respective 95% confidence intervals. The data was analyzed using the software R, v. 3.4.1.

The present study was approved by the ethics in research committee of the Universidade Federal de São Paulo and HEMVNC, with due authorization to waive the informed consent form of all patients.

## Results

Regarding the obstetric conditions at hospitalization, Table 1 illustrates the mean gestational age–which was 38 weeks–while the mean number of pregnancies and deliveries was 2.4 and 1.01, respectively. The median was 39 weeks regarding the gestational age, with the highest age being 41 weeks, and the lowest being 22. Among the patients, 48.1% were nulliparous, and 30.8% of pregnant women had already had at least one previous cesarean section. At least 6 antenatal care visits were performed by 89.4% of patients.

**Table 1:** Obstetric conditions at hospitalization of patients who underwent the B-Lynch technique

Condition	n	%	Average	95% CI	Median	Minimum value	Maximum value
Gestational age			38.0	37.4–38.6	39.0	22.0	41.0
≥ 37	86	82.7%					
34–36	11	10.6%					
≤ 33	7	6.7%					
TOTAL	104	100.0%					
Number of pregnancies*			2.4	2.0–2.7	2	1	9
1	44	42.3%					
2–4	48	46.2%					
≥ 5	12	11.5%					
TOTAL	104	100.0%					
Parity**			1.01	0.80–1.4	1	zero	6
Nulliparous	50	48.1%					
1–2	40	38.4%					
3–4	9	8.7%					
≥ 5	5	4.8%					
TOTAL	104	100.0%					
Previous cesarean section ( *n* = 104)	32	30.8%					
Antenatal care (≥ 6 visits) ( *n* = 104)	93	89.4%					

* Number of pregnancies: considered the sum of current and previous pregnancies, regardless of gestational age, vitality, or location.

** Parity: defined as the number of previous deliveries whose newborn weighed ≥ 500 g or reached gestational age ≥ 20 weeks, regardless of the number of fetuses or outcomes.

The perinatal characteristics of the patients are shown in Table 2 , with 96.2% having a cesarean delivery, with 93.3% of these having cephalic presentation. Regarding birth weight, 13% were underweight, and 9.2% were macrosomal. Table 2 also shows that 100% of patients received oxytocin and misoprostol in the pharmacological treatment for atony, and 86.5% also used ergometrine.

**Table 2:** Perinatal characteristics of patients who underwent the B-Lynch technique

Condition	n	%
Type of delivery		
Cesarean section	100	96.2
Vaginal	4	3.8
TOTAL	104	100.0
Presentation		
Cephalic	97	93.3
Pelvic	7	6.7
TOTAL	104	100.0
Birth weight (g)		
< 2,500	14	13.0
2,500–3,999	84	77.8
≥ 4,000	10	9.2
TOTAL	108	100.0
Use of oxytocin to treat PPH		
Yes	104	100.0
No	0	0.00
TOTAL	104	100.0
Use of ergomethrin		
Yes	90	86.5
No	14	13.5
TOTAL	104	100.0
Use of misoprostol		
Yes	104	100
No	0	0.00
TOTAL	104	100
Days of postpartum hospitalization		
≤ 3	88	84.6
4–7	15	14.4
≥ 8	1	1.0
TOTAL	104	100.0

Table 3 shows the percentage of patients who presented some risk factor for UA.

**Table 3:** Risk factors for uterine atony identified in patients who underwent the B-Lynch technique

Risk factor	n	%
NONE ( *n* = 104)	38	36.5
Previous cesarean section ( *n* = 104)	32	30.8
Use of oxytocin for induction/conduction of labor ( *n* = 104)	17	16.3
Preeclampsia ( *n* = 104)	12	11.6
Fetal macrosomia ( *n* = 104)	9	8.7
Placental abruption ( *n* = 104)	9	8.7
Twinning ( *n* = 104)	4	3.8
Grand multiparity ( *n* = 104)	5	4.8
Polyhydramnios ( *n* = 104)	2	1.9

The frequency of complications after the surgery is analyzed and shown in Table 4 , and 77.9% had no complications related to B-Lynch suture or PPH. Regarding hospitalization, most patients remained hospitalized for up to 3 days, and 15.4% required intensive care unit (ICU) care. The same table also shows that the frequency of puerperal infection was 1%, and there were five hysterectomies due to failure of hemostasis with the use of the B-Lynch technique representing 4.8% of patients.

**Table 4:** Puerperal complications in patients who underwent the B-Lynch technique

Complications	n	%
One or more complications ( *n* = 104)	23	22.1
Blood transfusion ( *n* = 104)	14	13.5
ICU admission ( *n* = 104)	16	15.4
Puerperal infection ( *n* = 104)	1	1.0
Surgical site infection ( *n* = 104)	1	1.0
Puerperal hysterectomy ( *n* = 104)	5	4.8

## Discussion

The B-Lynch hemostatic suture was included in the clinical practice protocol of the HMEVNC in 2005. Since then, the clinical experience at this Maternity Hospital School has only improved. 16

A limitation of the present study should be noted. The patients were observed in a short period, which makes it impossible to assess late complications. A total of 104 patients were divided into groups of pregnancy-puerperium cycle variables. Regarding gestational age, we had 82.7% of full-term births, and 17.3% of prematurity, with 61.2% of these being late preterm babies. Prematurity is often associated with indications intended to safeguard the mother-fetus binomial. 24 The premature interruption of high-risk pregnancies often has the cesarean section as the mode of delivery, and this association implies a greater hemorrhagic risk. The lower uterine segment becomes thicker, and has a larger surface area of the myometrium, which would lead to greater blood loss. 25

Among the patients, 48.1% were nulliparous, and only 5.8% were considered grand multiparous women. There is a direct relation between socioeconomic conditions and parity, as this condition is implicit in the access to information, as well as in the various methods for adequate family planning. Grand multiparity is an additional risk factor for worse mother-fetus outcomes, regardless of other factors. 22

At least 30.8% of pregnant women in our study had previously had a cesarean section. In 2016, we had 55% of cesarean deliveries in Brazil, whereas the rate was 40% in Brazil's unified health system (SUS), which is still far from the WHO recommendations. The main objective of the cesarean section is to safeguard the mother-fetus well-being in risk situation for this binomial. However, it is currently an iatrogenic epidemic in our country, with this important surgery included in the category of risk factor, among which we include the puerperal hemorrhages. 26

Regarding antenatal care, 89.4% had at least 6 visits. According to the Ministry of Health of Brazil, this number of appointments in a well-qualified manner and with professional training could be sufficient to identify risks and take preventive actions, or even to refer the patient to more complex services, thereby reducing the mother-fetus morbidity and mortality. 27

Concerning the clinical and obstetric conditions of patients at the time of admission, we found 36.5% of them had no risk factor for UA. Many cases do not present any identifiable risk factor in the antepartum nor in the intrapartum, which justifies a careful observation of the risk signs of hemodynamic instability so that the hemorrhagic control actions can be performed immediately. 28

In relation to the perinatal characteristics of the patients studied, 96.2% had a cesarean delivery with precise obstetric indications, and this type of operative delivery represents an increased hemorrhagic risk. 26 29

Still regarding the perinatal characteristics, we show that 100% of the patients received oxytocin and misoprostol in the pharmacological treatment for atony, and 86.5% also used ergometrine, showing that all patients received the appropriate pharmacological treatment to correct UA, and that 13.5% did not receive ergometrine because they had chronic hypertension or preeclampsia, which contraindicates the use of ergotamine derivatives. 7

Among the various risk factors for PPH, previous cesarean section stood out in the present study, representing 30.8% of patients, followed by the use of oxytocin, which corresponded to 16.3%, and 11.6% were diagnosed with preeclampsia. The alarming rates of cesarean sections are going up worldwide, and the increase in hemorrhagic risk is well established both in patients with a previous cesarean section who underwent a new cesarean section and in patients who are undergoing cesarean delivery for the first time. 26 The use of oxytocin may pose greater risks for the development of UA. In 2010, Balki et al. demonstrated that there would be a desensitization of specific uterine oxytocin receptors for patients who were exposed to high doses of oxytocin for a long period, which would justify its refractoriness in previously exposed patients. 30

I emphasize that 15.4% needed ICU hospitalization, and 13.5% were transfused with blood products. It would be reasonable to assume that even though the percentage of patients in the ICU and transfused is high, it could have been even higher if the patients had not undergone the B-Lynch technique. It is still valid to state that the obstetric emergency related to hemorrhage itself poses a risk for transfusion. It is noteworthy that the uterine blood flow is ∼ 600 ml/minute in a term pregnancy, which can lead to a very considerable blood loss in a short period. 31

The frequency of puerperal infection and surgical wound was only 1%. Puerperal hysterectomy is considered a failure of the method, and it occurred in 4.8% of patients, showing that the vast majority was preserved from a procedure with greater comorbidity in addition to having their uterus preserved. In addition to the B-Lynch technique being simple and of lower risk for comorbidities, the preservation of the uterus as a way of preserving fertility had a great impact on patients and family members. It is noteworthy that, in this study, 95.2% of the patients had their uterus preserved, escaping the possibility of having a more aggressive procedure and with a greater comorbidity such as hysterectomy.

However, it should be noted that individual training to perform the B-Lynch technique and the preparation of multiprofessional teams for immediate intervention in the control of PPH are mandatory for the results to be satisfactory.

Although our results were very satisfactory to recommend its practice, we understand that there is room for assessing the possible long-term impacts, and that they were not included in the objectives of the present study.

More studies are necessary in our country to clarify the role of B-Lynch in the women's reproductive health including fertility, chronic pelvic pain, dyspareunia, and menstruation.

## Conclusion

The clinical experience with the B-Lynch technique was satisfactory since it presented few complications, with excellent results in hemorrhagic control. Previous cesarean section, the use of oxytocin and preeclampsia stood out as factors related to the indication of the application of the technique, and the success rate in controlling PPH was 95.2%.
